# Percutaneous closure of patent foramen ovale for paradoxical embolism in acute limb ischemia

**DOI:** 10.1007/s12928-018-0542-9

**Published:** 2018-08-14

**Authors:** Hiroya Takafuji, Shinobu Hosokawa, Riyo Ogura, Yoshikazu Hiasa

**Affiliations:** Department of Cardiology, Tokushima Red Cross Hospital, 103 Irinokuchi, Komatsushima-cho, Komatsushima, Tokushima 773-8502 Japan

**Fig. 1:**
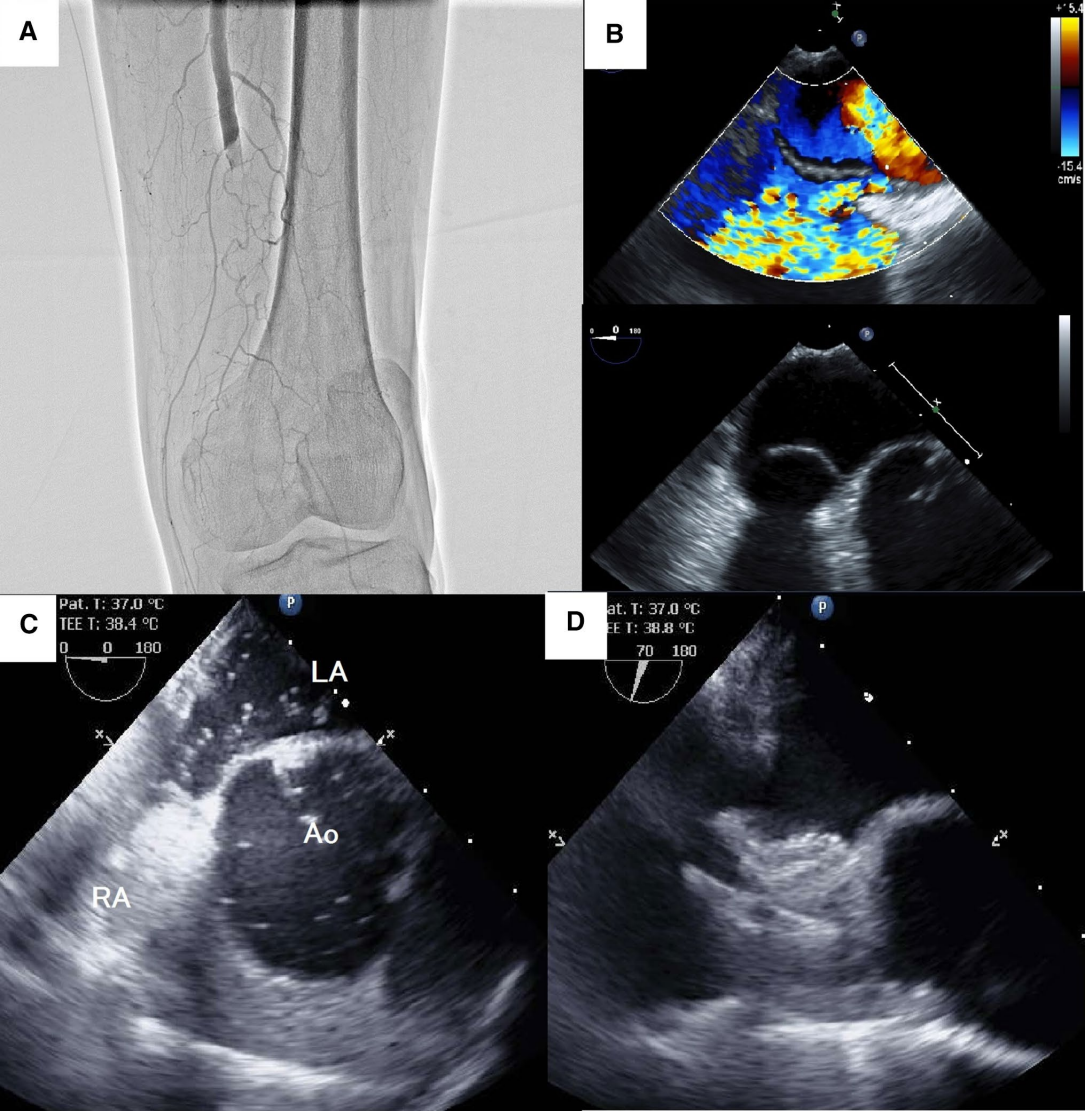
**a** Angiography of the left leg revealed thrombus in the left superficial femoral artery. **b** Transesophageal echocardiography showed atrial septal aneurysm and bidirectional shunt between the right and left atrium. **c** Transesophageal echocardiography showed large number of microbubbles through the patent foramen ovale. **d** Transesophageal echocardiography showed an Amplatzer Cribriform deployed. *LA* left atrium, *RA* right atrium, *Ao* aorta

A 67-year-old man was admitted to our hospital for acute left leg pain and numbness. He had a history of gastric cancer. However, he had no history of hypertension, diabetes, or dyslipidemia. On examination, the left leg was cold and pale and the left popliteal and posterior tibial arteries were not palpable. Electrocardiogram showed normal sinus rhythm. Duplex ultrasonography revealed a thrombus that was occluding the left superficial femoral artery (SFA) that resulted in absence of Doppler signals. Acute limb ischemia (ALI) was suspected; angiography confirmed thrombotic occlusion of the left SFA at the distal part without atherosclerotic plaque or dissection (Fig. 1a). The patient underwent successful thromboembolectomy via a Fogarty catheter. In transesophageal echocardiography (TEE), there was no evidence of other embolic sources such as atrial appendage thrombus, appendage stasis with reduced flow velocities, low ejection fraction, or aortic arch atherosclerotic plaques. However, patent foramen ovale (PFO) with continuous bidirectional shunt and atrial septal aneurysm were detected (Fig. 1b). Furthermore, an agitated saline bubble test with Valsalva maneuver identified significantly large numbers of microbubbles through the PFO (Fig. 1c). Venous echocardiography showed deep venous thrombosis in the left popliteal vein. Based on these findings, ALI was considered to have occurred because of paradoxical embolism from the vein to the artery. Percutaneous closure of PFO with a 25-mm Amplatzer Cribriform (Abbott, Chicago, Illinois) was performed, because sizing balloon measured the stretched diameter of PFO was 7.2 mm by TEE (Fig. 1d). After procedure, the patient received aspirin 100 mg/day and direct oral anticoagulant (DOAC) for the first 6 months, followed by DOAC alone. Recurrent embolic events have not been documented.

There has been an increasing interest in the association between PFO and stroke. In addition, PFO is also associated with systemic embolism; coronary, renal, spleen and peripheral arteries [1, 2]. However, paradoxical embolism is diagnosed by exclusion. Therefore, definite diagnosis is confirmed by existence of PFO without other reason. For that reason, it is important to do careful examination of embolic source. Percutaneous PFO closure is feasible and effective for patients with PFO to prevent recurrence of embolic events.
